# Early radiographic osseointegration of a novel highly porous 3D-printed titanium collar for megaprostheses compared to a previous generation smooth HA-coated collar

**DOI:** 10.1007/s00402-022-04760-3

**Published:** 2023-01-04

**Authors:** Thomas Haider, Iosif Pagkalos, Guy Morris, Michael C. Parry, Lee M. Jeys

**Affiliations:** 1 Department of Orthopaedic Oncology, Royal Orthopaedic Hospital Birmingham, Birmingham, UK; 2 Department of Orthopaedics and Trauma Surgery, Medical University of Vienna/General Hospital of Vienna, Waehringer Guertel 18-20, 1090 Vienna, Austria; 3 Faculty of Health and Life Sciences, Aston University, Birmingham, UK

**Keywords:** Megaprosthesis, Orthopaedic oncology, Revision arthroplasty, Endoprosthetic replacement

## Abstract

**Purpose:**

Extracortical osseointegration at the collar-bone interface of megaprostheses is associated with improved implant stability, lower rates of stem fracture and loosening. The use of hydroxy-apatite (HA-) coated collars showed mixed results in previously published reports. A novel collar system has recently become available utilizing additive manufacturing technology to create a highly porous titanium collar with a calcium-phosphate coated surface. The aim of this study was to evaluate our early experience with this novel collar and compare it to the previously used HA-coated model.

**Methods:**

Twenty patients who underwent megaprostheses implantation utilizing the novel collar system were case matched to 20 patients who had previously undergone a HA-coated collar. A minimum radiological follow-up of three months was available in all included patients. Osseointegration was evaluated using postoperative plain radiographs in two planes based on a previously published semi-quantitative score.

**Results:**

Compared to the HA-coated collar the use of the novel highly porous collar was associated with a higher proportion of cases demonstrating osseointegration at the bone-collar interface (80% vs. 65%). Application of the highly porous collar led to a significantly shortened time to reach the final ongrowth score (173 ± 89 days vs. 299 ± 165 days, *p < *0.05). At one year follow-up, 90% of the novel collars had reached their final osseoingration grade compared to 50% in the HA-coated collar group (*p < *0.001). Radiological osseointegration was seen in 71% for highly porous collars where the indication was revision arthroplasty, compared to 27% in reported in the literature.

**Conclusion:**

These results indicate more reliable and accelerated osseointegration at the bone-collar interface of a novel highly porous collar system compared to a previously used HA-coated collar. Further studies are warranted to confirm these findings.

## Introduction

In complex revision arthroplasty and orthopaedic oncology, a commonly used mode of reconstruction is endoprosthetic replacement using megaprostheses [1, 2]. Despite recent advancements in implant design and refinements of implantation techniques resulting in improved prosthesis survival rates, tumour prosthesis remain associated with high complication and revision rates [1, 2]. Available literature demonstrates all-cause revision rates of more than 40% in the first 10 years after implantation [1]. Particularly aseptic loosening was repeatedly reported to be a common mode of failure and cause for revision surgery [2–5]. One study reported around 30% of patients with tumour prosthesis required at least one revision due to aseptic loosening within 15 years after surgery [1]. Extramedullary osseointegration at the bone-endoprosthetic interface was shown to provide advantageous biomechanical properties thus potentially reducing the risk for aseptic loosening, stem fracture and periprosthetic fractures [3, 6–9].

In an effort to improve ongrowth capacity at the bone-prosthesis interface different techniques have been employed with mixed results. Aside from bone grafting around the prosthesis shoulder coating of the prosthesis collar with hydroxy-apatite (HA) was utilized [2, 3, 6, 10–12]. A recent study observed osseointegration around HA-coated collars in megaprostheses in less than half of reviewed cases [2]. Previous implant failure due to aseptic loosening, arguably the indication that would benefit most from collar osseointegration, was associated with an even worse ongrowth rate with the percentage dropping down to 27% [2]. Evolution of additive manufacturing technology not only allowed for emergence of custom-made patient-specific implants but also facilitated the incorporation of highly complex materials and surfaces in implant design and production processes [13, 14]. Particularly in cases of severe bone loss around the acetabulum but also in complex knee revision arthroplasty highly porous augments and cones, respectively, have recently gained popularity due its usability and reliabilty [14, 15].

We recently started using a highly porous calcium-phosphate coated EPORE^®^ collar with tumour prostheses (Implantcast, Buxtehude, Germany). EPORE^®^ utilizes tiny rods of Titanium (~ 350 μm) which are arranged together in an open stochastic random pattern to form ‘pores’ of 100–500 μm, similar to that of trabecular bone.

Therefore, the aim of this study was to report on our experience with this novel collar and evaluate the radiological follow-up for evidence of osseointegration.

## Methods

### Patients

**Fig. 1:**
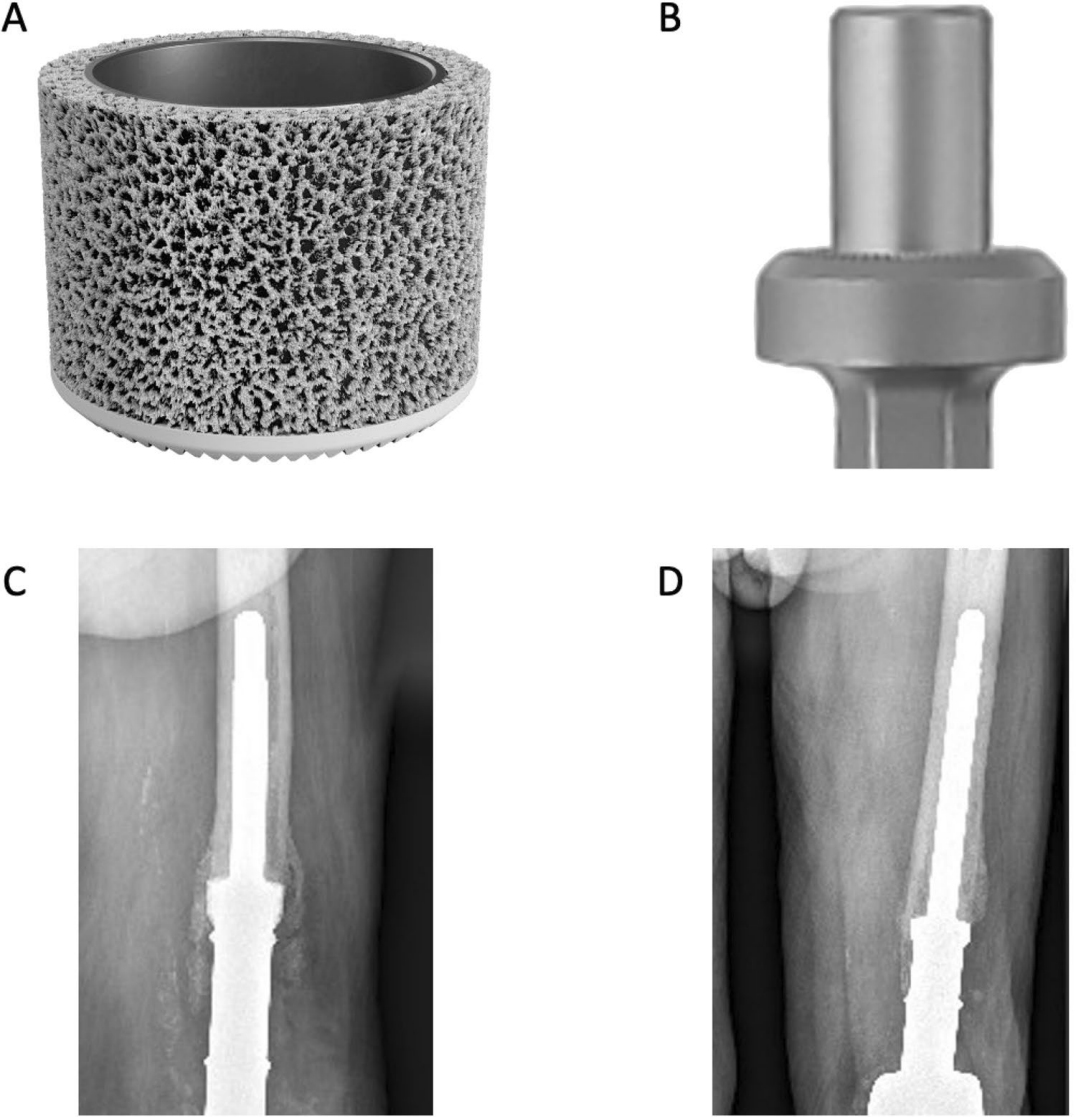
Photographs of the 3D-printed highly porous collar (**A**) and hydroxy-apatite coated collar (**B**). Radiological appearance of the highly porous (**C**) and the hydroxy-apatite coated collar (**D**)

In a first step, we retrospectively identified cases in which the novel 3D-printed collar (EPORE^®^, Implantcast, Buxtehude, Germany) was utilized. Patient with a radiological follow-up including x-rays in two planes of at least three months after implantation were included in this study. Subsequently, the case matched series of cases of smooth HA-coated collars (MUTARS^®^, Implantcast, Buxtehude, Germany) with the same requirements on available radiological follow-up was included. Both collars are depicted in Fig. 1. In all cases, fellowship-trained orthopaedic surgeons with longstanding experience in the use of megaprostheses for oncology and revision arthroplasty performed the surgery. The included patients underwent reconstruction utilizing either proximal or distal femur endoprosthetic replacements (MUTARS^®^, Implantcast, Buxtehude, Germany). Uncemented stems were HA-coated in all cases.

### Radiological evaluation of bone ongrowth

Osseointegration was graded using a previously published semi-quantitative scale [2]. Bone ongrowth was assessed by analysing two bone-collar interfaces on the ap (anteroposterior) and lateral x-rays, respectively. The final available set of x-rays was graded. Time to final ongrowth was assessed by identifying the first available postoperative radiographs with the final ongrowth grade. All cases were independently evaluated by two raters and consensus was reached in all cases. Grade 1 represents no visible ongrowth on all four interfaces, grade 2 indicates bony overgrowth with gap formation, grade 3 equals osseointegration in one or two interfaces and grade 4 means visible ongrowth on a least three interfaces [2]. Clinical records were analysed and variables including revision surgeries and complications were collected.

### Statistics

Nominal variables between two groups were compared with the Fisher’s exact test, while the chi-square test was utilized for comparison among three or more groups. The Mann–Whitney *U*-test was utilized to compare continuous variables between two groups. For calculating the time to final ongrowth, the Kaplan–Meier methods was used and curves were compared using the log-rank test. Cases without any radiological evidence for osseointegration were censored at final available follow-up. Spearman’s rank correlation coefficient was calculated for age and ongrowth score as well as age and time to final ongrowth. Age groups with cut-offs of 40 and 65 years were selected for subgroup analysis because they could prove to be potentially clinically relevant. A *p* value of < 0.05 was considered statistically significant for all employed tests. Data were analysed and visualized using SPSS (Version 25.0, IBM, Armonk, NY, USA) and GraphPad Prism (Version 9, GraphPad Software, La Jolla California USA). Data are given as mean ± standard deviation (SD) if not stated otherwise.

## Results

We included 40 patients with 20 patients in each cohort. The mean age was 63 (± 18) years for the entire study population of which 57.5% (*n = *23) were male. Detailed demographics for both study groups are depicted in Table 1. There were no statistically significant differences in demographics between the HA-collar and 3D-printed collar group. As we started using the 3D-printed collar only in 2020, the mean follow-up was significantly longer in the HA-coated collar cohort as expected (568 ± 315 vs. 248 ± 125 days, *p < *0.001). In all 3D-printed collar cases cement fixation of the stem was used compared to 60% in the HA-collar group (*p < *0.01). Indications in both groups included revision arthroplasty, periprosthetic joint infections, primary bone tumours and bone metastases and were comparable among both groups. However, revision for aseptic loosening as indication for surgery was more common in cases where a 3D-printed collar was used (20% vs. 5%, *p = *0.34). In each group, there was a single case of stem loosening of a cemented stem due to persisting infection requiring further surgical revision (Table 2).

**Table 1:** Demographic characteristics of both study cohorts

	Overall (*n = *40)	HA-coated (*n = *20)	3D-printed (*n = *20)	*p* value
Female/male, *n* (%)	17 (42.5) / 23 (57.5)	12 (60) / 8 (40)	5 (25) / 15 (75)	0.054
Age, mean (range)	63 (17–83)	61 (17–83)	65 (19–82)	0.495
Type of implant	–	–	–	0.752
Proximal femur EPR, *n* (%)	20 (50)	11 (55)	9 (45)	–
Distal femur EPR, *n* (%)	20 (50)	9 (45)	11 (55)	–
Follow-up in days, mean (SD)	408 (± 287)	568 (± 315)	248 (± 125)	** < 0.001**
Cement fixation, *n* (%)	32 (80)	12 (60)	20 (100)	** < 0.01**
Previous revision surgery, *n* (%)	27 (67.5)	12 (60)	15 (75)	0.501
Perioperative chemotherapy, *n* (%)	4 (10%)	2 (10)	2 (10)	1
Indication		–	–	0.7
Revision arthroplasty, *n* (%)	13 (32.5)	5 (25)	8 (40)	–
Periprosthetic infection, *n* (%)	14 (35)	7 (35)	7 (35)	–
Primary malignancy, *n* (%)	10 (25)	6 (30)	4 (20)	–
Bone metastasis, *n* (%)	3 (7.5)	2 (10)	1 (5)	–
Osseointegration yes/no, *n* (%)	29 (72.5)	13 (65) / 7 (35)	16 (80) / 4 (20)	0.48
Ongrowth score		–	–	0.693
1, *n* (%)	9 (22.5)	6 (30)	3 (15)	–
2, *n* (%)	2 (5)	1 (5)	1 (5)	–
3, *n* (%)	19 (47.5)	9 (45)	10 (50)	–
4, *n* (%)	10 (25)	4 (20)	6 (30)	–
Days to final ongrowth, mean (SD)	230 (± 142)	299 (± 165)	173 (± 89)	** < 0.05**
Stem loosening, *n* (%)	2 (5)	1 (5)	1 (5)	1

Bold *p* value < 0.05

*EPR* endoprosthetic replacement, *SD* standard deviation

**Table 2:** Comparison of demographic characteristics of patients with and without osseointegration

	No osseointegration (*n = *11)	Osseointegration (*n = *29)	*p* value
Female/male, *n* (%)	4 (36.4) / 7 (63.6)	13 (44.8) / 16 (55.2)	0.73
Age, mean (range)	59 (17–83)	65 (29–83)	0.473
Type of implant	–	–	0.48
Proximal femur EPR, *n* (%)	7 (63.6)	13 (44.8)	–
Distal femur EPR, *n* (%)	4 (36.4)	16 (55.2)	–
Follow-up in days, mean (SD)	332 (± 218)	437 (± 308)	0.402
Cement fixation, *n* (%)	8 (72.7)	24 (82.8)	0.66
Previous revision surgery, *n* (%)	6 (54.5)	21 (72.4)	0.451
Perioperative chemotherapy, *n* (%)	2 (18.2)	2 (6.9)	0.3
Indication	–	–	0.546
Revision arthroplasty, *n* (%)	4 (36.4)	9 (31)	–
Periprosthetic infection, *n* (%)	2 (18.2)	12 (41.4)	–
Primary malignancy, *n* (%)	4 (36.4)	6 (20.7)	–
Bone metastasis, *n* (%)	1 (9.1)	2 (6.9)	–
Stem loosening, *n* (%)	1 (9.1)	1 (3.4)	0.479

*EPR* endoprosthetic replacement, *SD* standard deviation

**Fig. 2:**
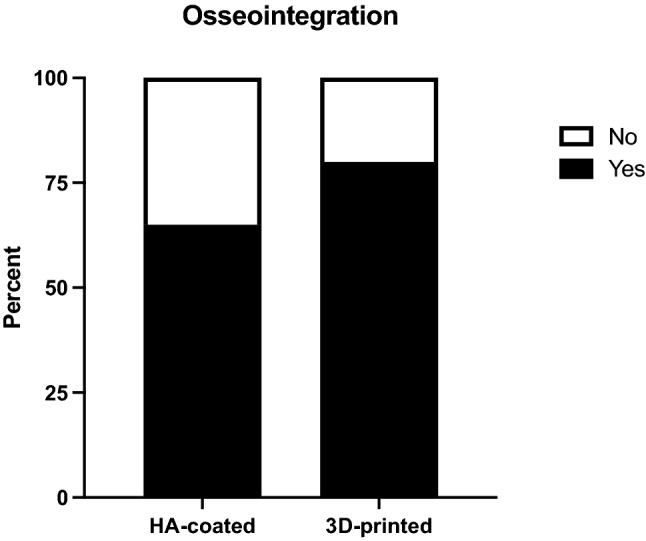
Bar chart comparing percentages of patients with and without osseointegration between both study groups. *HA* hydroxy-apatite

**Fig. 3:**
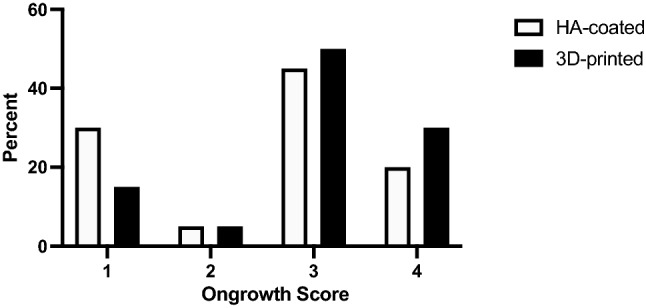
Distribution of ongrowth scores among both study groups. *HA* hydroxy-apatite

**Fig. 4:**
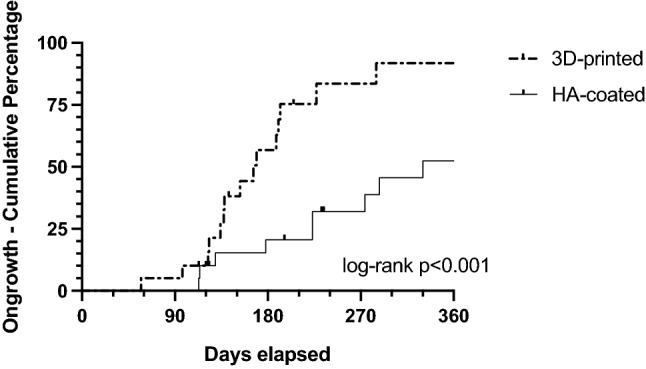
Kaplan–Meier curve comparing the cumulative incidence of final ongrowth score between both study cohorts during the first year of follow-up. *HA* hydroxy-apatite

Overall, in 29 patients (72.5%) radiological evidence for osseointegration was observed with a higher percentage found in the 3D-collar group. This difference, however, did not reach statistical significance (80% vs. 65%, *p = *0.48, Fig. 2). There were more grade 1 cases in the HA-collar cohort while grade 3 and grade 4 cases were more prevalent in the 3D-printed collar group (Fig. 3). Patients in the 3D-collar cohort reached their final ongrowth score faster when compared to the HA-collar cohort (173 ± 89 days vs. 299 ± 165 days, *p < *0.05). This difference was confirmed in the Kaplan–Meier analysis. At one year follow-up 90% of 3D-printed collars showed final ongrowth score compared to 50% of HA-coated collars (Fig. 4, log-rank test *p < *0.001).

We further sought to compare cemented and uncemented stem fixation. In 75% of cemented stems collar osseointegration was observed compared to 62.5% (*p = *0.66). Also, time to final ongrowth score was lower in the cemented cohort (217 ± 127 vs. 300 ± 205 days, *p = *0.62). However, both differences did not reach statistical significance.

There was no significant correlation between age and final ongrowth score (Spearman’s *ρ* = 0.238, *p = *0.07). Also, when dividing the entire cohort in patients under and above 40 years, no significant differences in ongrowth proportions were found. The same was true when moving the cut-off to 65 years. There was a weak correlation between age and time to final ongrowth indicating reduced time to osseointegration with younger age (Spearman’s *ρ* = 0.412, *p < *0.05). The proportion of cases with osseointegration was not significantly different between cases of revision surgery and primary implantation (77.8% vs. 61.5%, *p = *0.239). Also, when comparing proximal and distal femur endoprosthetic replacements no significant difference in percentage of osseointegration was found (65% vs. 80%, *p = *0.48). All five cases revised due to aseptic loosening showed ongrowth on at least one bone-collar interface.

## Discussion

To the best of our knowledge, this is the first study to report on the osseointegrative capacity of a novel highly porous-coated collars around megaprostheses. These implants are often utilized in highly complex cases of limb reconstruction including revision arthroplasty and oncological cases with an inherent high complication risk. Addition of a highly porous and calcium-phosphate coated collar at the bone-implant interface is thought to facilitate osseointegration. This extracortical bridge between the implant and bone increases implant stability and is able to lower peak forces at the intramedullary site of primary fixation consequently reducing the risk for aseptic loosening and periprosthetic fractures [2, 3, 6–9]. Also, preventing intraarticular debris from entering the intramedullary bone-implant surface (“purse-string effect”) has been brought forward as potential benefit of additional bone overgrowth at the bone-implant interface [12, 16].

Aseptic loosening was previously shown to be one of the major causes for revision in megaprostheses [1]. This complication was shown to develop particularly within the first five years after surgery [11]. Also, undertaking a revision due to aseptic loosening was reported to be associated with a lower probability of finding radiological evidence for osseointegration [2].

We found two cases (2.5%) of stem loosening, one in each cohort, both on the background of a periprosthetic joint infection requiring further surgical interventions, due to infection. However, no case of aseptic loosening was identified in either group at a mean follow-up of approximately one year.

The indication for the original surgery in five cases (12.5%) was aseptic loosening and in four of these cases the novel 3D-printed collar was utilized. In all five patients, (100%) bone ongrowth at the collar-bone interface was evident radiologically, compared to a reported rate of 27% by Davies et al. This finding potentially indicates that the unique structure of this collar is more likely to provide an osteoconductive and osteoinductive microenvironment even in cases of previous aseptic loosening compared to other collar designs.

In our series, previous revision surgery was not associated with lower rates of osseointegration but, interestingly, rather the opposite was observed with 77.8% osseointegration in previously revised patients compared to 61.5% in primary cases, again in contrast with a previous report from Davies et al. [2] As details regarding the number and indications of previous revision surgeries were not available for this study, a high heterogeneity of these variables might have potentially biassed this result. There were more patients with at least one prior revision in the 3D-printed collar group. Also, with more cases of revision arthroplasty in this group (8 vs. 5 cases) the prerequisites for osseointegration, based on the previous report by Davies et al. were worse compared to the HA-collar group. We argue that these findings further underline the capacity of the 3D-printed collar to induce bone ongrowth even in cases of potentially impaired local biological conditions.

The highly porous structure used in the here evaluated novel 3D-printed collar resembles cancellous bone architecture which aims for mechanically relevant bone ingrowth. The unique microstructure creates a capillary effect at the surface and in combination with calcium-phosphate coating is thought to be particularly instrumental for providing a osteoinductive and osteoconductive milieu [14]. Highly porous surfaces were previously reported to be associated with favourable clinical and radiological outcome in complex reconstruction of the acetabulum and revision knee arthroplasty [14, 15]. But also in megaprostheses porous coating at the implant shoulder was previously associated with high rates of osseointegration and low rates of aseptic loosening. Chao et al. found extracortical bone coverage at the coated implant area of more than 75% on average and less than 5% of cases showed stem loosening [6]. However, their technique involved bone grafting, in most cases autologous graft, around the implant shoulder thus limiting comparability to our series as no local adjuvants were used. Interestingly, cement fixation was associated with lower coverage and ongrowth thickness compared to uncemented fixation in their series [6]. In contrast, cement fixation was associated with a higher rate of osseointegration (75% vs. 62.5%) and reduced time to final ongrowth score in this study. During implantation we paid utmost attention to prevent cement entrapment at the collar-bone interface which might serve as a possible explanation for improved results with cemented stem fixation in our series.

Particularly in uncemented megaprostheses aseptic loosening was shown to develop early, within the first 5 years [11]. The novel 3D-printed collar was associated with an expedited osseointegration when compared to the previously used HA-coated collar (173 ± 89 days vs. 299 ± 165 days, *p < *0.05). Therefore, we suggest that particularly in uncemented revision situation where an uncemented stem fixation is preferred or the only option, for instance due to specific anatomical situations or sclerotic endosteal bone on the basis of previous cement fixation, the highly porous collar might prove most beneficial. In our series, most implants were cemented and all prostheses in the 3D-printed collar group were cemented. Chao et al. reported increased bone ongrowth with uncemented stems compared to cement fixation [6].

Follow-up, in general, was limited by the retrospective character of this study. As expected due to later introduction of the 3D-printed collar, mean follow-up was significantly shorter for this cohort. Chao et al. reported that final bone ongrowth was observable at the two-year mark in most cases with only a few included cases showcasing further bone ongrowth beyond this point [6]. Longer follow-up potentially would have further improved results for the novel collar system.

Davies et al. reported a high rate of grade 2, that is overgrowth with an apparent diastasis between new bone formation and the collar, of 41% while only in one patient in our series (2.5%) this pattern was evident [2].

Previous histological workup of explanted megaprostheses revealed novel lamellar bone formation around HA-coated collars while no bone interface in prostheses without collar was reported [3]. On the contrary, Tanzer et al. did not observe bone ingrowth at the porous-coated shoulder of explanted prostheses histologically but fibrous tissue was found which is also capable of providing additional stability [10, 17]. Preclinical studies have shown that additive-manufactured titanium, as also used in the here studied 3D-printed collars, promotes novel bone formation and exhibits biomechanical properties comparable to cancellous bone [18–20].

In summary, our findings demonstrate a higher rate of radiological evidence for osseointegration when utilizing a novel 3D-printed collar for megaprostheses. Compared to the HA-coated collar final ongrowth was reached significantly faster. To confirm these results further studies are required.

## Limitations

We acknowledge that this study has several limitations. As pointed out by Tanzer et al. reliability of plain radiographs to accurately determine bone ongrowth is questionable [17]. Also, only a semi-quantitative evaluation of collar ongrowth with inherent shortcomings was utilized. However, given the fact that radiological evidence of osseointegration at the bone-collar interface was previously unanimously reported to be associated with lower rates of stem loosening indicates that radiographic evidence for ongrowth is clinically relevant. Also, the available timepoints showed a high inter- and intragroup heterogeneity due to the retrospective design of this study. Heterogeneity of available time points and stem fixation between the two study groups might have influenced the results. Also, smoking was previously demonstrated to negatively impact collar-bone ongrowth. Unfortunately, information about the smoking status of included patients was not available for analysis in this study. Long-term studies will be required to evaluate whether the observed findings translate into favourable outcome in regards to implant survival and revision rates. Also, no functional outcome was available for patients included in this study. Nonetheless, we are confident that novel insights provided by this study are clinically relevant and of interest in view of challenges associated with utilizing megaprostheses.

## Data Availability

Data will be made available upon request to the corresponding author.
